# Enhancing Centelloside Production in *Centella asiatica* Hairy Root Lines through Metabolic Engineering of Triterpene Biosynthetic Pathway Early Genes

**DOI:** 10.3390/plants12193363

**Published:** 2023-09-23

**Authors:** Miguel Angel Alcalde, Javier Palazon, Mercedes Bonfill, Diego Hidalgo-Martinez

**Affiliations:** 1 Department of Biology, Healthcare and the Environment, Faculty of Pharmacy and Food Sciences, University of Barcelona, 08028 Barcelona, Spain; miguel.psr.94@gmail.com (M.A.A.); mbonfill@ub.edu (M.B.); 2 Biotechnology, Health and Education Research Group, Posgraduate School, Cesar Vallejo University, Trujillo 13001, Peru

**Keywords:** metabolic engineering, centelloside, triterpene, synthetic biology, machine learning, plant biotechnology

## Abstract

*Centella asiatica* is a medicinal plant with a rich tradition of use for its therapeutic properties. Among its bioactive compounds are centellosides, a group of triterpenoid secondary metabolites whose potent pharmacological activities have attracted significant attention. Metabolic engineering has emerged as a powerful biotechnological tool to enhance the production of target compounds. In this study, we explored the effects of overexpressing the squalene synthase (*SQS*) gene and transcription factor *TSAR2* on various aspects of *C. asiatica* hairy root lines: the expression level of centelloside biosynthetic genes, morphological traits, as well as squalene, phytosterol, and centelloside content. Three distinct categories of transformed lines were obtained: LS, harboring *At-SQS*; LT, overexpressing *TSAR2*; and LST, simultaneously carrying both transgenes. These lines displayed noticeable alterations in morphological traits, including changes in branching rate and biomass production. Furthermore, we observed that the expression of T-DNA genes, particularly *aux2* and *rolC* genes, significantly modulated the expression of pivotal genes involved in centelloside biosynthesis. Notably, the LS lines boasted an elevated centelloside content but concurrently displayed reduced phytosterol content, a finding that underscores the intriguing antagonistic relationship between phytosterol and triterpene pathways. Additionally, the inverse correlation between the centelloside content and morphological growth values observed in LS lines was countered by the action of *TSAR2* in the LST and LT lines. This difference could be attributed to the simultaneous increase in the phytosterol content in the *TSAR2*-expressing lines, as these compounds are closely linked to root development. Overall, these discoveries offer valuable information for the biotechnological application of *C. asiatica* hairy roots and their potential to increase centelloside production.

## 1. Introduction

*Centella asiatica*, commonly known as *Gotu Kola*, is a renowned medicinal plant with a rich history of traditional use in various cultures for its therapeutic properties [1]. Among its numerous plant secondary bioactive compounds are centellosides, a group of triterpenes that have attracted considerable attention due to their potent pharmacological activities, including anti-inflammatory, antioxidant, and wound-healing effects. The high medicinal value of centellosides has prompted extensive research aimed at understanding their biosynthesis and developing strategies to enhance their production [2].

The biosynthetic pathways of secondary metabolites, including centellosides, are complex and tightly regulated processes. In plants, secondary metabolite production is influenced by various factors, such as gene expression, enzymatic activity, and metabolic flux. Among the key regulatory components are phytosterols; these primary metabolites and membrane constituents serve as precursors for various hormones and play a crucial role in determining the production of specific triterpenes. It has been observed that when sufficient phytosterols have been synthesized to meet the plant’s growth requirements, the biosynthesis of triterpenes takes precedence [3,4].

Metabolic engineering has emerged as a powerful biotechnological tool to manipulate the biosynthetic pathways of secondary metabolites in plants. By introducing specific genes into plant cells, researchers can modulate gene expression and metabolic pathways to enhance the production of desired compounds [5,6]. In the context of centellosides, studies have focused on manipulating key biosynthetic genes to augment their yields in *C. asiatica*.

One such gene of interest encodes squalene synthase (*SQS*), a key enzyme in the biosynthesis of triterpenes [4,7]. By overexpressing the *SQS* gene, it is possible to increase levels of the precursor squalene, potentially leading to higher centelloside production. The engineering of secondary metabolite production also involves transcription factors (TS) [8,9]. For example, *TSAR2* (triterpenoid saponin activating regulator 2) has been identified as having a direct effect on the expression of the *HMGR* gene, which encodes 3-hydroxy-3-methylglutaryl-coenzyme A reductase, an important enzyme in the triterpene biosynthetic pathway [10,11,12]. The overexpression of the *HMGR* gene has shown promising results in increasing phytosterol and triterpenoid content in different plant species [11,13,14].

Hence, the generation of *C. asiatica* hairy roots harboring *SQS* and *TSAR2* offers a potential opportunity to enhance centelloside yields. In this study, we explored the impact of these genetic modifications on the expression of centelloside pathway genes and morphological traits in different *C. asiatica* hairy root lines. Additionally, we examined the correlation between squalene, phytosterol, and centelloside contents in the transformed roots, aiming to elucidate the regulatory mechanisms underlying centelloside biosynthesis in this medicinal plant.

The findings from this study could offer valuable insights into the biotechnological potential of *C. asiatica* hairy root lines and pave the way for novel approaches to enhance centelloside production. Ultimately, these advancements could provide the pharmaceutical and cosmeceutical industries with a sustainable source of centellosides and help to unlock their therapeutic potential for various health applications.

## 2. Results

### 2.1. Integration of Transgenes by PCR

In the present study, we obtained four different types of hairy root lines: LS, carrying the exogenous *SQS* gene from *A. thaliana*; LT, which contains the transcription factor *TSAR2*; LST, with both of the aforementioned genes; and L1, utilized as the control. These hairy root lines harbored all the *rol* and *aux* genes from the pRi plasmid of *R. rhizogenes*.

Genomic DNAs from transformants and *C. asiatica* leaves were used for PCR analysis of multiple lines (Figure S1). Initially, the *rolC* gene served as a positive control to confirm the transformed nature of the obtained roots, given its involvement in hairy root formation. Following three rounds of selection with cefotaxime, the *VirD* gene was utilized to verify the absence of *R. rhizogenes* in the transformants, as this gene is present in the bacterial plasmid but not in the T-DNA region integrated in plant cells. The results demonstrated the presence of the *rolC* gene in *R. rhizogenes* (positive control) and the transformants but not in *C. asiatica* leaves (negative control). Conversely, the *VirD* gene was absent in all the lines. Finally, the presence of the target genes, *At-SQS* and *TSAR2*, was verified, and the following lines were selected for further analysis: LST(1-3), LS(1-3), and LT(1-3).

### 2.2. Influence of Transgenes on Endogenous Gene Expression

**Figure 1:**
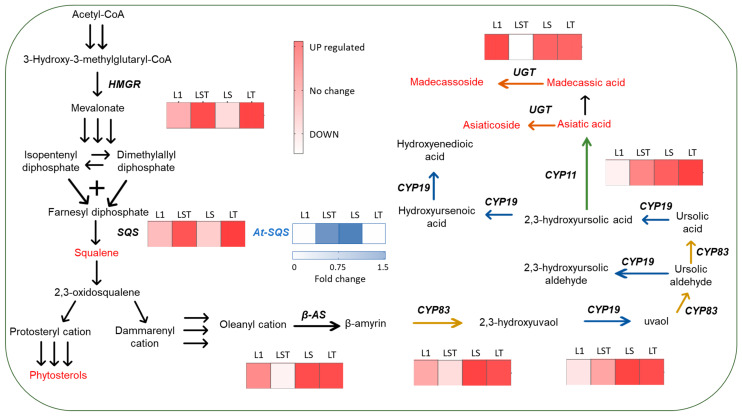
Overview of the centelloside biosynthetic pathway and gene expression profile. L1 denotes the control line, while LST, LS, and LT represent the transformed lines. The reddish heatmap indicates the upregulation (UP) or downregulation (DOWN) of gene expression for *HMGR* (encoding the 3-hydroxy-3-methylglutaryl coenzyme A reductase), *SQS* (encoding squalene synthase), *β-AS* (encoding beta amyrin synthase), *CYP83* (cytochrome *CYP716A83*), *CYP19* (cytochrome *CYP714E19*), *CYP11* (cytochrome *CYP716C11*), and *UGT* (*UGT73AD1*). The bluish heatmap reflects the fold change in gene expression of *At-SQS* (squalene synthase from *Arabidopsis thaliana*).

By conducting a genetic expression analysis, we explored the impact of the *At-SQS* and *TSAR2* transgenes on the centelloside biosynthetic genes. The results, illustrated in Figure 1, depict the expression profiles of the targeted endogenous genes.

The expression of the HMGR and SQS genes differed notably among the examined lines. Specifically, lines overexpressing TSAR2 exhibited significantly elevated expression levels of these two endogenous genes compared with the control. A correlation analysis revealed a strong positive correlation (r = 0.98) between HMGR and SQS, suggesting that TSAR2 enhances the expression of these genes.

Surprisingly, this transcription factor exhibited no remarkable effects on or correlations with other genes involved in centelloside biosynthesis. In addition, the transformants exclusively overexpressing At-SQS had gene expression values on par with those of the control line. Consequently, no conspicuous augmentations or detrimental influences on the expression of the endogenous genes within the pathway were observable.

To examine the impact of the *rol* and *aux* transgenes derived from the pRi plasmid of *R. rhizogenes* on the centelloside biosynthetic genes, we generated multiple regression models using the randomForestSRC R-package (see Section 4). The models treated the endogenous genes *HMGR*, *SQS*, *β-AS*, *CYP83*, *CYP19*, *CYP11*, and *UGT* as dependent variables, whereas the expression levels of *rolA*, *rolB*, *rolC*, *aux1*, and *aux2* genes were considered independent variables. Among these models, the one pertaining to *CYP11* exhibited the highest R-squared value (0.7349), indicating the strongest level of fit. Conversely, the *SQS* gene model had the smallest R-squared value (0.1387). This suggests that the expression levels of the pRi plasmid transgenes are either unconnected with or exert a very limited influence on the expression behavior of the *SQS* gene.

**Figure 2:**
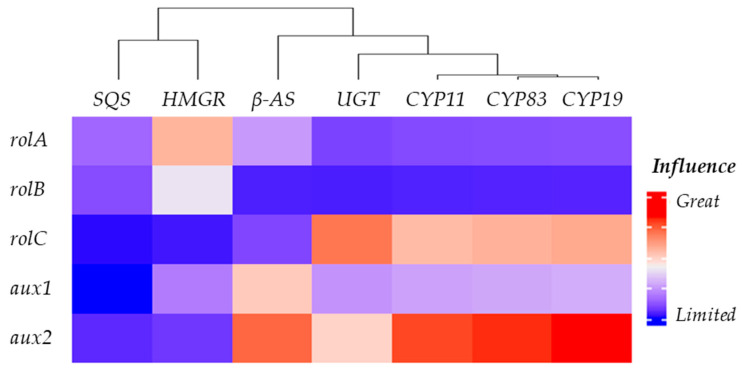
Comprehensive heatmap illustrating the impact of *rol* and *aux* genes on the endogenous gene expression profile in *Centella asiatica* hairy roots.

The variable importance metric was utilized to compare the impact of pRi plasmid transgenes on the profile of each endogenous gene, aiding in the interpretation of the multivariate regression models. For a comprehensive understanding of the influence of transgene expression, Figure 2 presents a cluster analysis of the pathway genes.

Remarkably, *aux2* exhibited a favorable impact on *β-AS*, *CYP83*, *CYP19*, and *CYP11*, with *UGT* displaying the least susceptibility and *CYP19* showing the most pronounced effect. Following closely, *rolC* emerged as the subsequent influential factor, specifically influencing *CYP83, CYP19, CYP11*, and *UGT* compared with *aux2*. The influence of *rolC* was particularly prominent on the *UGT* gene. The *aux1* gene, on the other hand, was notable solely for its impact on *β-AS*, while *rolA* exhibited only a marginal effect on *HMGR*. Among the endogenous genes, *SQS* was the least susceptible to the influence of the *rol* or *aux* genes, closely followed by *HMGR*.

### 2.3. Sterols, Squalene, and Centolloside Profiles in Response to Transgenes TSAR2 and At-SQS

Through a comprehensive phytochemical analysis, we examined the effects of the At-*SQS* and *TSAR2* transgenes on centelloside, squalene, and total sterol profiles. Sterols constitute key compounds within a competitive pathway toward centellosides, while squalene serves as a common precursor.

**Figure 3:**
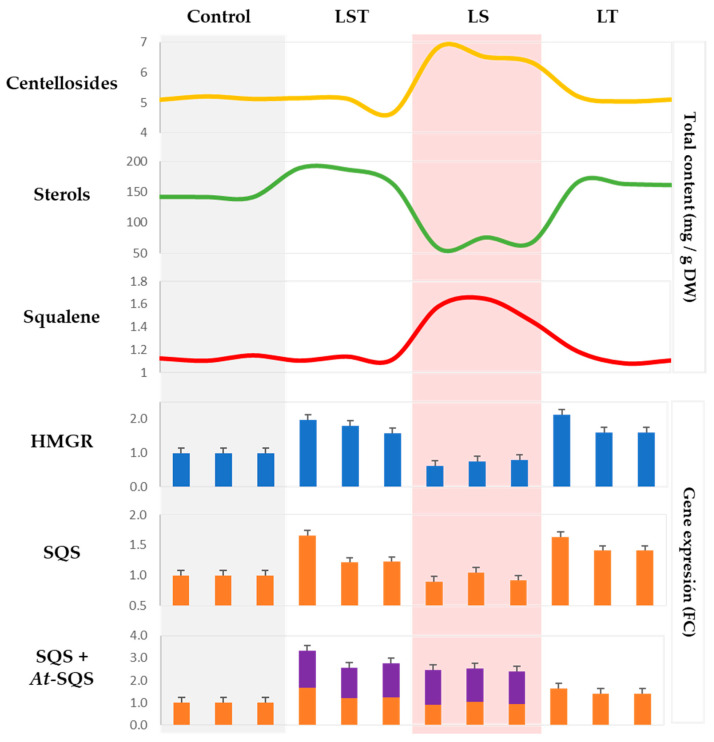
Comparative plot of various types of hairy root lines. The density plot illustrates the profiles of sterols, squalene, and centellosides, while the bar plots display the gene expression profiles of *HMGR, SQS,* and *At-SQS* in terms of fold change values. The stacked column highlighted in purple represents the contribution of *At-SQS*. See Table S1 for more details.

In Figure 3, the upper section illustrates the total content (measured in mg/g DW) of centellosides, sterols, and squalene in the four types of hairy root lines. Notably, the LS lines exhibited the highest centelloside content (6.56 ± 0.26 mg/g DW), differing significantly from the other lines (Figure S2). The direct influence of the *At-SQS* transgene on production levels was evident.

Greater variability was observed in the sterol content, which ranged from 188.31 to 56.88 mg/g DW among the root lines. Specifically, the LST lines displayed a significant increase compared with the control, whereas a pronounced decrease was observed in the LS lines, resulting in values lower than the control. Conversely, the values of the LT lines did not differ significantly from those of the LST lines. Noteworthy in this context was the clear positive effect of the transcription factor on sterol content, as well as on the *HMGR* gene, as previously mentioned.

The profile of the squalene content was similar to that of centellosides, with a significant increase in the LS lines (1.56 ± 0.10 mg/g DW), and nearly constant values in the other three lines. Accumulated expression values of native *SQS* and *At-SQS* genes in the LS line clearly demonstrated the positive impact of the *At-SQS* transgene on centelloside production. However, this positive effect was only observed in the absence of the transcription factor, suggesting that the activation of the sterol biosynthetic pathway effectively utilized the additional squalene generated by the heterologous enzyme.

### 2.4. Influence of Squalene and Sterol Content on Morphological Traits

**Figure 4:**
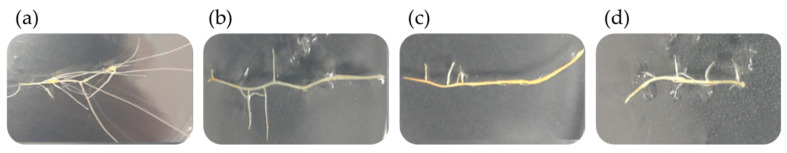
Average morphology of different hairy root lines of *Centella asiatica* at day 28 of growth: (**a**) control, (**b**) LST, (**c**) LS, (**d**) LT. The initial inoculum was a small section of 10 mg of fresh weight.

In this study, we deemed the branching rate, growth rate, and biomass productivity to be pertinent morphological traits owing to their biotechnological implications. The branching rate denotes the number of lateral roots per centimeter, growth rate signifies the elongation of roots in millimeters per day, and biomass productivity quantifies the daily biomass yield for each line. Various morphologies of hairy root lines after 28 days of growth are illustrated in Figure 4 and Figure S3.

**Figure 5:**
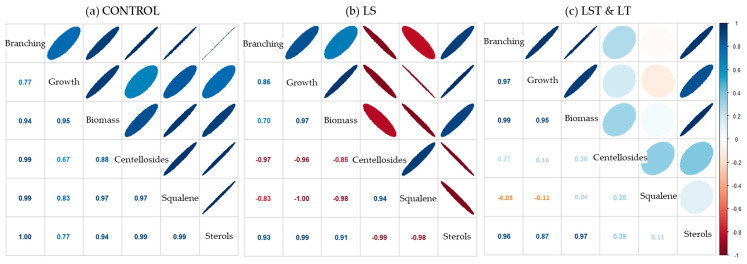
Pearson’s correlation analysis of morphological traits and metabolite content. (**a**) Correlation plot for the control line; (**b**) correlation plot for the LS lines; (**c**) correlation plot for the lines overexpressing the transcription factor. Values indicate the Pearson correlation coefficient (r).

By conducting a thorough correlation analysis between the morphological traits and the contents of squalene and sterols, we have unveiled a compelling depiction of how the accrual or depletion of these two distinct compounds intricately interweaves with the measured characteristics (Figure 5). The control line maintained a robustly positive correlation between the variables. However, in the LS line, which yielded significantly diminished levels of sterols yet substantially elevated quantities of squalene and, therefore, centellosides, a different pattern was discernible. Here, it became distinctly apparent that the aggregation of squalene exerted a deleterious impact on the underlying morphological traits.

Intriguingly, in the LST line, this adverse effect appeared to be counterbalanced by the presence of the transcription factor. This regulatory entity seemed to orchestrate a harmonizing influence, fostering amplified sterol production while concurrently addressing the extra squalene availability. This bifaceted mechanism effectively overcame the negative impact observed in the LS line, thus indicating the intricate interplay between genetic modulation and metabolite accumulation.

## 3. Discussion

This study further elucidates the complex interplay between specific genes and the centelloside biosynthetic pathway. Notably, the *C. asiatica* hairy root lines harboring *TSAR2* exhibited a substantial upregulation of the endogenous genes *SQS* and *HMGR*, suggesting they play pivotal roles in the regulation of centelloside biosynthesis. These findings align with those of Xu et al. [12], who observed increased *HMGR* gene expression in *Ganoderma lucidum* overexpressing the *GlbHLH5* transcription factor under methyl jasmonate elicitation, and Mertens et al. [11], who reported higher transcript levels of the *HMGR* gene in *Medicago truncatula* hairy roots overexpressing *TSAR2*. Moreover, instances of transcription-factor-mediated modulation of *SQS* gene homolog expression, leading to activated triterpenoid biosynthesis, have been observed in various species such as *Ziziphus jujuba* and *Torreya grand* [15,16].

A key factor in triterpenoid structural diversity is the cytochrome P450 family, particularly the CYP716 enzymes. These versatile enzymes, distributed widely across diverse plant species, have the capacity to catalyze triterpene oxidation at various positions [17]. Among the ensemble of genes, *CYP16C11* emerges as a significant player and is modulated by the insertion of T-DNA genes (Figure 2). Our findings are in parallel to those of Pandey et al. [18], who investigated the complex interactions of *CYP716C55* gene expression and metabolic pathways under Ri-T-DNA-mediated insertional mutagenesis, unraveling the intricacies of gene modulation within the dynamic landscape of *Ocimum* species hairy roots.

The *aux2* gene (1398 bp), a component of TR-DNA, plays a pivotal role in the induction and continuation of hairy root growth [19,20]. This oncogene orchestrates the conversion of naphthalene acetamide into the potent auxin naphthalene acetic acid, a crucial stage in hairy root development [21]. Notably, Srivastava et al. [22] revealed the significant influence of the *aux2* gene on 3-indol acetic acid (IAA) levels, morphological traits, and metabolite content in *Ocimum basilicum* hairy roots, with implications mirroring our own results, as its overexpression had a favorable impact on the expression of *β-AS, CYP,* and *UGT* genes.

By boosting beta-glucosidase activity, the *rolC* gene provides a gateway for the release of active cytokinins from their inactive counterparts [23]. Its versatile effects include roles in nicotine production in *Nicotiana tabacum* hairy roots [24], ginsenoside synthesis in *Panax ginseng* hairy roots [25], and resveratrol production in transgenic *Vitis amurensis* cell cultures [26]. In our study, we observed how the *rolC* gene positively influences *CYP* and *UGT* gene expression. Similarly, Inyushkina [27] noted that *rolC* gene expression led to increased transcript levels of *CYP98A3* subfamily members, crucial genes in rosmarinic acid biosynthesis.

The important role of the *rolA* gene in triterpenoid production has been reported in *Artemisia dubia* hairy roots, where it was found to directly enhance artemisinin production [28]. Interestingly, although the influence of the *rolA* gene in secondary metabolism remains less explored compared with other oncogenes, our work reveals its notable impact on the early triterpene pathway gene *HMGR*, which encodes an enzyme catalyzing vital steps in IPP biosynthesis [29].

The LST and LT lines stood out for their high phytosterol content, a direct effect of *TSAR2*. The influence of other transcription factors such as *WRKY* or *MYC2* (*WsWRKY1* and *WsMYC2*) on phytosterol biosynthesis has been demonstrated in studies on *Withania somnifera* [30,31].

The LS lines exhibited the highest squalene content, mirroring their centelloside content (Figure 3), which illustrates the effect of introducing the *SQS* gene from *A. thaliana* into the *C. asiatica* hairy root lines. This phenomenon has been previously studied in *Medicago truncatula* [32], *P. ginseng* [4], *W. somnifera* [33], *Taraxacum koksaghyz* [34], and *Eleutherococcus senticosus* [35], the results attesting to the intriguing connection between squalene synthase overexpression and enhanced triterpene levels.

As well as the highest centelloside content, the LS lines possessed the lowest phytosterol content (Figure 3), which reflects the fact that triterpene biosynthesis occurs once sufficient phytosterol has been synthesized, signaling the end of growth [3,4]. Supporting this observation, studies have shown that inhibiting the phytosterol biosynthetic pathway by antisense cycloartenol synthase led to a notable increase in ginsenosides (triterpenes) in *P. Ginseng* hairy roots [13]. A similar antagonistic interaction between the triterpene and sterol pathways has been observed in transgenic *Buplerum falcatum* roots [36] and in methyl-jasmonate-elicited *C. asiatica* plants, where free sterol decreased while centellosides increased [37].

Furthermore, it has been reported that triterpenic saponins can also contribute to root growth, as demonstrated in the case of a γ-pyronyl triterpenoid saponin, known as chromosaponin 1 in *Lactuca sativa* [38]. In *A. thaliana*, this compound interacts with the *aux1* protein, regulating the root gravitropic response [39].

Regarding the hairy root morphological traits, the strongest positive correlation between the different growth values with metabolite composition was observed in the control lines (Figure 5a). Conversely, the traits of LS lines exhibited an opposing trend (Figure 5b), which implies that the accumulation of squalene and subsequent elevation in centelloside content functions as a trigger for secondary metabolism, marked by the cessation of cell division and growth [40]. Curiously, in the LST and LT lines, the negative correlation observed in the LS lines disappears (Figure 5c). This remarkable fact finds its origins in the insertion of *TSAR2*, which directly affects the *HMGR* gene and, quite possibly, genes of the sterol biosynthetic pathway, resulting in a higher phytosterol content. These effects of the transcription factor contrast with the findings of Hey et al. [14], who observed a 2.44-fold increase in sterol production in transformed *A. thaliana* compared with the control.

Our findings further highlight the regulatory interplay between the phytosterol and triterpene production pathways, along with their impacts on hairy root morphological traits. Future research should focus on refining the modulation of the transgenes to achieve a harmonious equilibrium between production levels and morphological attributes.

## 4. Materials and Methods

### 4.1. Establishment and Selection of C. asiatica Hairy Roots

The protocol followed in this study was based on Alcalde et al. [41] with slight modifications. In vitro cultures of *C. asiatica* plants were maintained on Murashige and Skoog (MS) medium supplemented with vitamins, 30% (*w*/*v*) sucrose, and 2.7% (*w*/*v*) gelrite in a controlled growth chamber. The growth chamber provided a constant temperature of 25 °C and a long-day photoperiod of 16 h of light followed by 8 h of darkness.

To initiate the hairy root induction process, leaf segments from the plantlets were wounded and infected with two distinct strains of *R. rhizogenes* A4. The first strain carried the binary plasmid pBI121_*At-SQS*, which encodes squalene synthase 1 from *A. thaliana* [42]. The second strain carried the plasmid pK7WG2D,1_*TSAR2*, encoding a transcription factor [43]. To achieve a double mutation, a simultaneous infection involving both strains was performed. Prior to infection, these bacterial strains were cultured at 25 °C on solid YEB medium for 48 h, supplemented with 100 mg/L of kanamycin and 50 mg/L of rifampicin.

After the infection, the wounded leaf segments were placed on solid MS medium enriched with 50 mg/L of acetosyringone. Up to four leaf segments were placed per plate, and the plates were then incubated in darkness at 25 °C for 2 days. Subsequently, the explants were transferred to fresh plates with solid MS medium containing 500 mg/L of cefotaxime and incubated at 25 °C for 4 weeks.

Following this incubation period, the roots that developed on the leaf segments were carefully isolated and transferred to new solid MS medium supplemented with 500 mg/L of cefotaxime and 0.1 mg/L of kanamycin. This step was repeated every 2 weeks over a period of about 2 months to eliminate any residual presence of *R. rhizogenes*. Robustly growing transgenic roots were identified by excising the top 5–7 cm of the root apex along with its lateral branches. These selected root lines were then cultured on fresh solid MS medium at a temperature of 25 °C in darkness, and subculturing of the transgenic root lines was carried out under the same conditions every 2 weeks.

### 4.2. DNA Isolation and Transgenic Confirmation by PCR Analysis

The DNA isolation protocol, adapted from Alcalde et al. [41], involved several steps. Initially, 200 mg of hairy root tissue was pulverized with liquid nitrogen and combined with 0.75 mL of extraction buffer (containing 50 mM EDTA pH 8.0, 100 mM Tris pH 8.0, and 500 mM NaCl), 0.6 μL of β-mercaptoethanol, and 50 μL of 20% sodium dodecyl sulfate (SDS). This mixture was incubated at 65 °C for 10 min, followed by the addition of 250 μL of 5 M potassium acetate. After an ice incubation for 20 min and centrifugation at 4 °C for 20 min at 9000 g, the supernatant was collected, mixed with 1 mL of isopropanol, and incubated at −20 °C for 1 h. The resulting pellet was centrifuged at 8000 g for 15 min and air-dried, then resuspended in 140 μL of T10E1 buffer (Tris 10 mM, EDTA 1 mM) and centrifuged for 10 min at 14,000 g. To the retained supernatant, 15 μL of 3 M sodium acetate and 100 μL of isopropanol were added. The resulting supernatant was again retained and centrifuged at 15,000 g for 10 min. The genomic DNA within the pellet was air-dried at 37 °C for 10 min and then resuspended in 30 μL of T10E1 buffer. Subsequently, 1 μL of RNAse (10 mg/mL) was added, and the purity of the DNA was assessed using a NanoDrop 2000 Spectrophotometer (Thermo Fisher Scientific, Waltham, MA, USA).

For the purpose of transgenic confirmation, transgenes from *R. rhizogenes* strains, including *rolC*, *At-SQS*, *TSAR2*, and *virD* genes, were amplified by PCR within 0.5 mL tubes. To each tube, 12.5 μL of Green taq polymerase, 1 μL of forward primer, 1 μL of reverse primer, 2 μL of DNA, and 8.5 μL of Milli-Q water were added. The specific primers used are detailed in Table S1. The PCR protocol comprised an initial cycle at 94 °C for 5 min, followed by 35 cycles at 94 °C for 1 min, 60 °C for 30 s, and 72 °C for 1 min, with a final extension step at 72 °C for 5 min. The size of the PCR products was determined using agarose gel electrophoresis (1%).

### 4.3. Analysis of Morphological Traits

To initiate the cultures, an inoculum of 10 mg of fresh weight (FW) was introduced for each hairy root line onto solid MS medium plates. These cultures were consistently maintained through two consecutive subcultures, with intervals of 2 weeks, at a temperature of 25 °C in a dark environment. This approach is in accordance with our established methodology from previous investigations [44].

Following this cultivation period, the assessment of growth parameters was carried out, with three replicates conducted for each distinct line. The evaluated growth parameters included the branching rate, quantified as the number of lateral roots per centimeter of the initial stem root (number of lateral roots/cm). Additionally, the growth rate was calculated by measuring the average length of the lateral roots (mm/day). Finally, biomass productivity was determined by dividing the difference between the final FW and the initial FW by the number of days the growth occurred (mg/day).

### 4.4. Quantification of Centelloside Production

The determination of centelloside production was conducted in accordance with the method outlined by Alcalde et al. [41]. Initially, 0.5 g of freeze-dried hairy root material (DW) was carefully weighed, followed by the addition of a 10 mL suspension of methanol:H_2_O (9:1). This suspension was then subjected to sonication for 1 h at room temperature to facilitate extraction. Subsequently, the suspension underwent centrifugation at 2000 g for 10 min to separate the solid and liquid phases. The supernatant obtained from this process was carefully separated, and the centrifugation step was repeated once more to ensure thorough extraction. The supernatants collected from the various samples were placed into porcelain mortars and evaporated at 38 °C for approximately 24 h. Following this, the resulting material was redissolved in 1 mL of methanol.

The methanolic extracts were subsequently filtered using a 0.22 μm filter for HPLC quantification of centellosides. The HPLC system consisted of a Waters 600 Controller pump, a Waters 717 Autosampler automatic injector, a Jasco variable-length (UV) 1570 detector, and Borwin data analysis software version 1.5. A Lichrospher 100 RP18 5 μm column (250 × 0.4 mm) was employed for gradient chromatography at room temperature following the method described by Alcalde et al. [41]. The mobile phase consisted of acetonitrile and ammonium phosphate (10 mM), acidified to a pH of 2.5 with orthophosphoric acid to enhance the definition of compound peaks. The flow rate was maintained at 1 mL/min, with an injection volume of 10 μL. The detector wavelength was set at 214 nm, 1.00 au/v, and the total run time was 45 min.

For the quantification of centellosides, namely asiatic acid, madecassic acid, asiaticoside, and madecassoside, calibration curves were established using standards of these four compounds. Calibration concentrations ranged from 10 to 500 ppm, with specific points at 10, 25, 50, 100, 250, and 500 ppm.

### 4.5. Expression Analysis of Centelloside Pathway Genes by RT-qPCR

Total RNA extraction was performed as in Alcalde et al. [41]. Trizol reagent was added to an Eppendorf tube with 200 mg of FW material and incubated for 20 min at room temperature. After shaking to mix the contents, the tubes were centrifuged at 7500 g for 10 min at 4 °C. The RNA pellet obtained was subjected to a single wash with 1 mL of 75% ethanol. After gentle shaking to mix the contents, the tubes were centrifuged at 7500 g for 5 min at 4 °C. Following removal of the supernatant, vacuum drying was employed for 10 min to desiccate the tubes. To dissolve the RNA samples, 20 μL of DEPC-treated water was utilized. Subsequent to extraction, RNA samples were assessed for purity and concentration using the NanoDrop 2000 Spectrophotometer (Thermo Fisher Scientific, Waltham, MA, USA).

The treatment with DNAse was administered using the total RNA as a template, employing a fixed concentration (2 μg of RNA). Additionally, the RNA integrity was confirmed via agarose gel electrophoresis. The synthesis of the first-strand cDNA was performed using the SuperScriptTM IV First-Strand Synthesis System (Invitrogen from Thermo Fisher Scientific, Waltham, MA, USA) with 2 μg of RNA, precisely following the manufacturer’s protocols.

After the design of primers via Primer3Plus software (version: 3.3.0) (Table S2), RT-qPCR assays were performed utilizing the QuantStudio3 System (Thermo Fisher Scientific, Waltham, MA, USA). These amplifications were conducted in 10 μL reaction volumes, consisting of 1 μL of first-strand cDNA (25 ng/μL), 2 μL of DEPC-treated water, 5 μL of iTaq Universal SYBR Green Supermix (Biorad, Hercules, CA, USA), and 1 μL of each specific primer at a concentration of 10 μM. The PCR conditions involved an initial phase at 95 °C for 30 s, followed by 40 cycles of 95 °C for 15 s and 60 °C for 30 s. To confirm the specificity of each primer pair, a melting curve analysis was performed, starting at 95 °C for 15 s, followed by a gradual increase from 60 to 95 °C at a rate of 0.1 °C/s, and ending with another step at 95 °C for 15 s.

### 4.6. Determination of Squalene Content

The quantification of squalene content was conducted following the methodologies of Shen et al. [45] and Rothblat et al. [46], with minor adjustments. In brief, the hairy roots were subjected to overnight drying in an oven set at 60 °C. For the subsequent steps, 200 mg of the dried sample was employed. Extraction was carried out within glass test tubes, utilizing 2 mL of n-hexane, and conducted in a supersonic water bath maintained at 40 °C for a duration of 30 min. The supernatants thus obtained were collected, and the extraction process was repeated once more to ensure thorough extraction. The pooled fractions were subjected to evaporation at 40 °C. Subsequently, 1 mL of concentrated sulfuric acid was added, and the mixture was subjected to a 5 min incubation in a water bath set at 70 °C. Then, 0.5 mL of 37% formaldehyde solution was gradually added, with the tubes being shaken for comprehensive mixing, and the mixture was placed in a water bath set at 95 °C for a 5 min period. Finally, to reach a final volume of 4 mL, 2.5 mL of glacial acetic acid was added promptly, and the mixture was meticulously mixed. After an incubation of 5 min, the absorbance at 400 nm was gauged using a UV2310 spectrophotometer (DINKO instruments, Barcelona Spain).

### 4.7. Determination of Phytosterol Content

The quantification of phytosterol content was conducted as in Araújo et al. [47], with minor modifications. For the preparation of the Liebermann–Burchard (LB) reagent, 50 mL of acetic anhydride was added to an amber glass bottle, which was maintained at a chilled temperature over ice. After a 30 min period, 5 mL of concentrated sulfuric acid was cautiously added to the bottle.

To establish the phytosterol reference curve, β-sitosterol was employed as the standard. Specifically, 10 mg of β-sitosterol was dissolved within 1 mL of chloroform, yielding a concentration of 10 mg/mL. Gradient concentrations spanning from 0.02 to 0.1 mg/mL of β-sitosterol were generated by diluting the stock solution with chloroform. Within the context of this reference curve, 0.5 mL of each concentration from the gradient was combined with 0.5 mL of LB reagent. Additionally, a blank solution comprising 0.5 mL of chloroform and 0.5 mL of LB reagent was prepared.

To determine the phytosterol content, 0.1 g of the dried sample material was mixed with 5 mL of chloroform within a glass tube. Subsequently, sonication was carried out for 30 min at room temperature. The supernatants thus obtained were collected, and the extraction process was repeated once more. The pooled fractions were subjected to evaporation at 40 °C, and the resulting residues were resuspended within 2 mL of chloroform. A mixture comprising 0.5 mL of the resuspension and 0.5 mL of LB reagent was prepared. The absorbance was measured at 625 nm, ensuring that this measurement was taken within a maximum of 5 min after the addition of LB reagent. For the purpose of comparison, a blank solution was generated, comprising 0.5 mL of chloroform and 0.5 mL of LB reagent.

### 4.8. Statistical Analysis and Machine Learning Models

Statistical analysis was performed using GraphPad Prism version 6.04 for Windows, developed by GraphPad Software in La Jolla, CA, USA. The data are presented as mean values with standard deviations. To conduct statistical comparisons, a multifactorial ANOVA analysis was employed, followed by Tukey’s multiple comparison tests. A significance level of *p* ≤ 0.05 was used to indicate statistical significance.

Machine learning models and Pearson correlation analysis were carried out using R Statistical Software (version 4.3.1) [48]. The randomForestSRC package [49] was employed to implement multivariate regression models. The coefficient of determination (R-squared) was used as a metric to evaluate the fitting of each model. The corrplot package [50] was utilized to compute the Pearson correlation coefficient (r). The reduced accuracy values of the multivariate multiple regression model were employed for hierarchical clustering analysis and were visually represented through a heatmap.

## 5. Conclusions

In this study, we explored the effects of *At-SQS* and *TSAR2* transgenes on the centelloside biosynthetic pathway, revealing distinct impacts on gene expression profiles. *TSAR2* overexpression notably elevated *HMGR* and endogenous *SQS* expression, demonstrating a strong positive correlation with these two genes. The transcription factor exhibited limited influence on other pathway genes, although its overexpression enhanced sterol production. Overexpression of *At-SQS* alone had minimal effects on endogenous genes but significantly increased the squalene content. Further investigation of pRi plasmid-derived *rol* and *aux* transgenes revealed a significant positive impact of *aux2* and *rolC* genes on centelloside pathway genes, whereas the influence of *aux1* and *rolA* was more restricted. The overarching theme from the findings of this study is the intricate interplay between genetic modulation and gene expression, shedding light on the complex mechanisms governing centelloside biosynthesis. Additionally, a comprehensive phytochemical analysis unveiled the interactions between *At-SQS* and *TSAR2* transgenes and centelloside, sterol, and squalene content. In general, the accumulation of squalene in the absence of *TSAR2* had a detrimental effect on the sterol content. Moreover, by measuring the branching rate, growth rate, and biomass productivity of the hairy roots, we revealed that these morphological traits of biotechnological importance are negatively correlated with squalene, whereas their improvement was strongly positively associated with sterol.

## Data Availability

Data are contained within the article and Supplementary Material.

## Supplementary Materials

The following supporting information can be downloaded at: https://www.mdpi.com/article/10.3390/plants12193363/s1, Figure S1: PCR analysis: detection of *SQS*, *TSAR2*, *rolC*, and *VirD* genes in different *C. asiatic* transgenic root lines, Figure S2: statistical differences between different types of lines, Figure S3: morphology of different transformed root lines of *C. asiatica* on day 14 of their growth, Table S1: quantitative data for gene expression and metabolite profiling, Table S2: primers used for confirmation of transformed roots, Table S3: primers used for gene expression analysis.
