# Supplementary material for: Enhancing Centelloside Production in Centella asiatica Hairy Root Lines through Metabolic Engineering of Triterpene Biosynthetic Pathway Early Genes
Source: Plants (Basel). 2023 Sep 23;12(19):3363. doi: 10.3390/plants12193363 (PMC10574710; doi:10.3390/plants12193363)
Supplement: Supplementary file 1 [file plants-12-03363-s001.zip › plants-2592223-supplementary.pdf]

Supplementary Material

Enhancing Centelloside Production in *Centella asiatica* Hairy Root Lines through Metabolic Engineering of Triterpene Biosynthetic Pathway Early Genes

Miguel Angel Alcalde[1,2], Javier Palazon[1*], Mercedes Bonfill[1], Diego Hidalgo-Martinez[1,*]

[1] Department of Biology, Healthcare and the Environment, Faculty of Pharmacy and Food Sciences, University of Barcelona, 08028 Barcelona, Spain; miguel.psr.94@gmail.com (M.A.); mbonfill@ub.edu (M.B.)

[2] Biotechnology, Health and Education Research Group, Posgraduate School, Cesar Vallejo University, Trujillo, Peru.

* Correspondence: dhidalgo@ub.edu (D.H.); javierpalazon@ub.edu (J.P.)

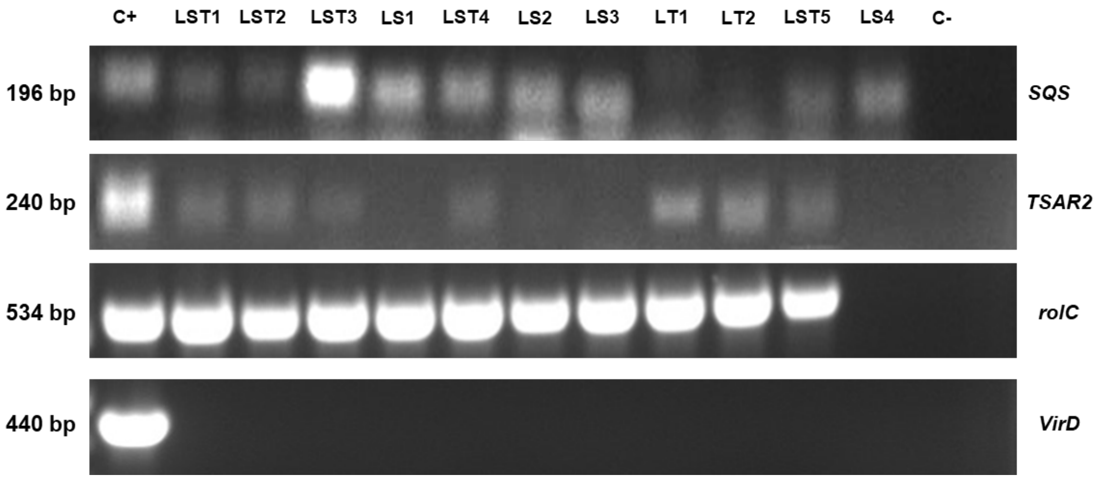

Figure S1. PCR analysis: detection of *SQS*, *TSAR2*, *rolC* and *VirD* genes in different *C. asiatica* transgenic root lines.

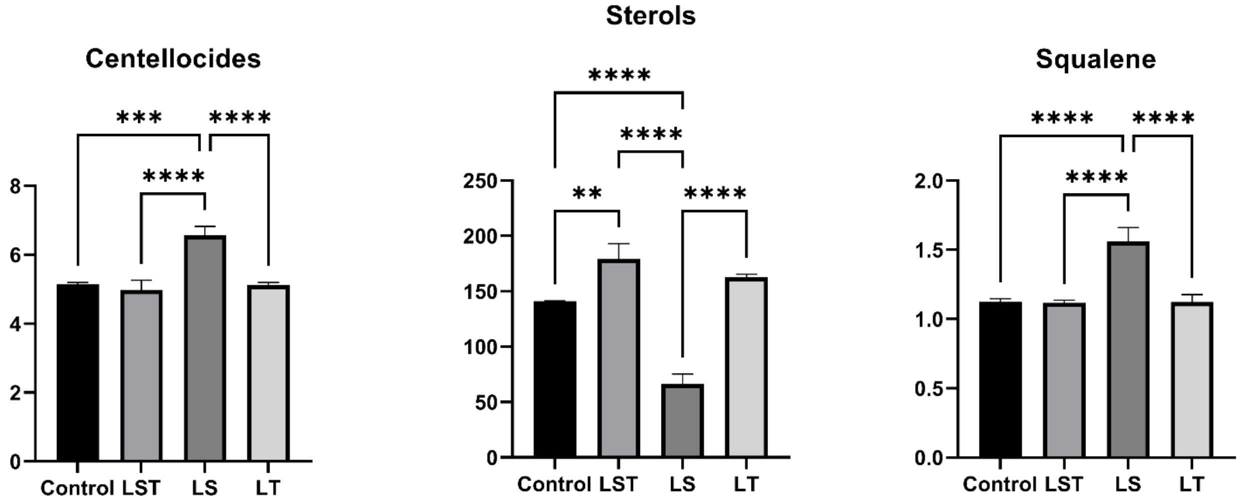

Figure S2. Statistical differences between different types of lines.

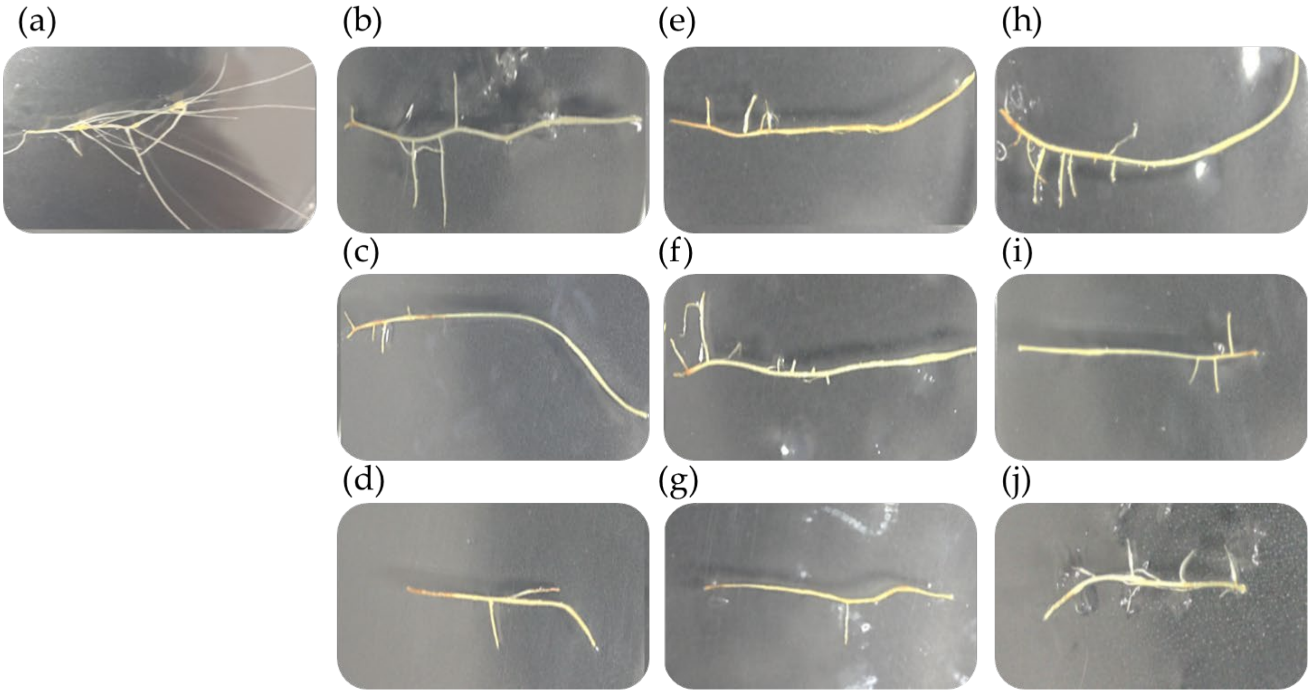

Figure S3. Morphology of different transformed root lines of *C. asiatica* on day 14 of their growth: (a) Control, (b) LST-1, (c) LST-2, (d) LST-3, (e) LS-1, (f) LS-2, (g) LS-3, (h) LT-1, (i) LT2 and (j) LT-3. The initial inoculum was a small section of 10 mg of fresh weight.

Table S1: Quantitative data for gene expression and metabolite profiling.

**Type of Line**	**Gene expression (fold change)**								**Metabolites**					
	HMGR		*SQS*		*At-SQS*		*SQS + At-SQS*		Centellosides content (mg/g DW)		Squalene content (mg/g dry weight)		Total Sterols (mg/g DW)	
	mean	SD	mean	SD	mean	SD	mean	SD	mean	SD	mean	SD	mean	SD
Control	1.00	0.00	1.00	0.00	0.00	0.00	1.00	0.00	5.14	0.06	1.12	0.02	141.12	0.23
LST	1.79	0.20	1.37	0.25	1.52	0.15	2.88	0.39	4.98	0.29	1.12	0.02	179.17	13.92
LST	0.73	0.09	0.96	0.08	1.50	0.04	2.46	0.07	6.56	0.26	1.56	0.10	66.20	9.12
LT	1.78	0.30	1.48	0.13	0.00	0.00	1.48	0.13	5.11	0.08	1.12	0.05	162.85	2.74

Table S2. Primers used for confirmation of transformed roots.

Gene	Primer sequence	Temperature melting (ºC)	Amplicon size (pb)	Accession number
rolC	F: TAACATGGCTGAAGACGACC<br>R: AAACTTGCACTCGCCATGCC	60	534	KX986281.1<br>(*R. rhizogenes* A4)
At-SQS	F: TGGGGAGCTTGGGGACGATGC<br>R: CGGCGTTACGGAGCTCGGTGTT	58	196	Mirjalili et al. [26] -<br>NM_119630.4<br>(*Arabidopsis thaliana*)
TSAR2	F: CCCCAAATTCATCCCCTACT<br>R: CAAGAGCTGCAAGAGCAATG	60	240	KR349466.1<br>(*Medicago truncatula*)
VirD	F: GGAGTCTTTCAGCATGGAGCAA<br>R: GGAGTCTTTCAGCATGGAGCAA	56	440	KX986281.1<br>(*R. rhizogenes* A4)

Table S3. Primers used for gene expression analysis.

Gene	Primer sequence	Temperature melting (ºC)	Amplicon size (pb)	Accession number
3-hydroxy-3-methylglutaryl coenzyme A reductase (HMGR)	F: CTCCTTCGTACTCCCTCGAGTC<br>R: CCCGTCCAGTGACTTTCCAG	60	101	KJ939450.2<br>(*Centella asiatica*)
Squalene synthase (SQS)	F: TGAGGAGAATTCGGTCAAGG<br>R: GCACAAAACCGGAAGATAGC	60	126	AY787628.1<br>(*Centella asiatica*)
beta-amyrin synthase (β-AS)	F: TTACGTTTGCTGGGAGAAGG<br>R: TATACCCCAAGAACCGCAAG	60	134	AY520818.1<br>(*Centella asiatica*)
CYP716A83 (CYP83)	F: TAGCTCTGCATGCACTTTCG<br>R: CCAGGTCCCTTTGATTTCAC	60	105	KU878849.1<br>(*Centella asiatica*)
CYP714E19 (CYP19)	F: AACCACACACACATCCTTGG<br>R: TCCTTTGATGAGCCCAAGAC	60	116	KU878852.1<br>(*Centella asiatica*)
CYP716C11 (CYP11)	F: CCCGATTCAACGACTCTTTC<br>R: CCGGTTGTTTGATCGACTTC	60	104	KU878852.1<br>(*Centella asiatica*)
UGT73AD1 (UGT)	F: TCTGGAAGCAGTTTGTGAGG<br>R: CGCCAATCTTCACTACATCG	59	101	KP195716.1<br>(*Centella asiatica*)
rolA	F: GAATGGCCCAGACCTTTGGA<br>R: TTGGTCAGGGAGGAAATCGC	60	116	EF433766.1<br>(*R. rhizogenes* A4)
rolB	F: CAACCGGATTTGGCCAGAGA<br>R: ATAGGGTTGCATCGTGGTCG	60	135	EF433766.1<br>(*R. rhizogenes* A4)
rolC	F: CGCGCTCATCACCAATCTTC<br>R: ACAGAAAGTGCGGCGAAGTA	60	135	EF433766.1<br>(*R. rhizogenes* A4)
aux1	F: TTCGAAGGAAGCTTGTCAGAA<br>R: CTTAAATCCGTGTGACCATAG	60	350	M61151.1<br>(*R. rhizogenes* A4)
aux2	F: GAACCACAAACCCAAGACGC<br>R: CTGTGGCGAAGTTGTTGCTC	60	125	M61151.1<br>(*R. rhizogenes* A4)
β-actin	F: TGACAATGGAACTGGAATGG<br>R: CAACAATACTGGGGAACACT	58	80	KF699319.1<br>(*Panax ginseng*)
